# Structure-based prediction reveals FAM105A as an African swine fever virus I215L interactor

**DOI:** 10.52601/bpr.2025.250057

**Published:** 2026-10-31

**Authors:** Xing Chen, Danyang Zhang, Yuxi Yang, Fayu Yang, Shilei Zhang

**Affiliations:** 1 State Key Laboratory for Animal Disease Control and Prevention, College of Veterinary Medicine, Lanzhou University, Lanzhou Veterinary Research Institute, Chinese Academy of Agricultural Sciences, Lanzhou 730046, China; 2 Gansu Province Research Center for Basic Disciplines of Pathogen Biology, Lanzhou Veterinary Research Institute, Chinese Academy of Agricultural Sciences, Lanzhou 730046, China; 3 African Swine Fever Regional Laboratory of China, Lanzhou Veterinary Research Institute, Chinese Academy of Agricultural Sciences, Lanzhou 730046, China

**Keywords:** African swine fever virus, I215L, FAM105A, Protein interaction, AlphaFold3

## Abstract

African swine fever virus (ASFV) causes a fatal hemorrhagic disease in pigs, leading to severe economic losses worldwide. The viral ubiquitin-conjugating enzyme I215L (E2) is critical for ASFV replication and modulation of host antiviral responses. Host deubiquitinating enzymes (DUBs) bind to E2 enzymes to inhibit the transfer of ubiquitin. In this study, we systematically screened 34 swine DUBs using HDOCK docking and AlphaFold3 modeling, identifying FAM105A as the most probable interactor. Co-immunoprecipitation assay validated the interaction between I215L and FAM105A. Structural analyses revealed seven I215L residues (E19, N20, M99, S102, A104, D108, S116) that form defined polar contacts, among which M99, D108, and S116 are critical for binding stability. These findings identify FAM105A as a novel host target of ASFV I215L, establishing a new paradigm for studying viral-host protein interactions.

## INTRODUCTION

African swine fever virus (ASFV) is the causative agent of African swine fever (ASF), a lethal hemorrhagic disease of domestic pigs and wild boars that poses a serious threat to global pig production. The ongoing spread of ASF across Europe and Asia has caused devastating economic losses (Dixon *et al.* 2020; Galindo and Alonso 2017). Due to the limited understanding of the ASFV-host interaction, particularly the interactions between viral proteins and host proteins, no effective vaccines or antiviral drugs are available (Zhang *et al.* 2023).

Ubiquitination is a fundamental post-translational modification regulating protein degradation, signaling pathways, and innate immunity (Song and Luo 2019). Manipulation of the ubiquitin system has emerged as a key viral strategy to promote replication and suppress host antiviral responses (Heaton *et al.* 2016). ASFV encodes I215L, a conserved viral ubiquitin-conjugating enzyme (E2) that is mono- and di-ubiquitinated, playing a critical role in the viral replication and immune evasion (Freitas *et al.* 2018). I215L inhibits type I interferon signaling by promoting degradation of STAT2, recruiting the E3 ligase RNF138 to block K63-linked ubiquitination of TBK1, and suppressing cGAS–STING signaling. I215L also interacts with the 40S ribosomal protein RPS23, translation initiation factor eIF4, and the cellular ligase Culin 4B to hijack the host translation machinery and regulate protein synthesis (Barrado-Gil *et al.* 2020; Huang *et al.* 2021; Riera *et al.* 2023).

Deubiquitination, which counterbalances ubiquitination, is mediated by deubiquitinating enzymes (DUBs), also known as ubiquitin thioesterases, which remove or edit ubiquitin chains to maintain cellular homeostasis (Isaacson and Ploegh 2009). Given their critical regulatory functions, identifying swine DUBs that interact with I215L is therefore an important step toward understanding how ASFV interacts with the host ubiquitin system. However, traditional strategies for protein-protein interaction analysis using mass spectrometry (MS) were costly and time-consuming. Recent advances in computational structural biology now enable accurate large-scale prediction of protein–protein interactions (Fenster *et al.* 2024; Zhang *et al.* 2024). Methods such as HDOCK docking and AlphaFold3 structure modeling offer powerful tools for probing virus–host protein interfaces, particularly in the absence of experimental data (Abramson *et al.* 2024; Yan *et al.* 2020). These approaches provide a novel strategy for identifying swine DUBs that interact with ASFV I215L.

In this study, we integrated HDOCK docking and AlphaFold3 structural modeling to screen swine DUBs for potential interactions with ASFV I215L. FAM105A was identified as the most probable interactor, and the interaction was experimentally validated by co-immunoprecipitation. Structural modeling and mutational scanning further delineated seven key I215L residues that stabilize the interaction. These findings advance our understanding of ASFV I215L-host interactions and may establish a new paradigm for studying viral-host protein interactions.

## MATERIALS AND METHODS

### Retrieval and selection of candidate proteins

Protein sequences of DUBs from *Sus scrofa* were retrieved from the UniProt database (https://www.uniprot.org; accessed July 2, 2025). A total of 456 sequences were obtained. Duplicates were removed, and when multiple entries shared the same protein name, those with reviewed annotations and available three-dimensional structures were preferentially retained. After filtering, 34 unique candidates were selected for subsequent analysis. The UniProt IDs, protein names, and sequence lengths of these 34 candidates are listed in supplementary Table S1.

### Prediction of protein–protein interactions

Potential interactions between ASFV I215L (PDB ID: 7WLH) and the 34 candidate proteins were assessed using HDOCK (http://hdock.phys.hust.edu.cn/) and AlphaFold3 (https://alphafoldserver.com/). For HDOCK, docking scores and confidence scores were generated for all candidates. Proteins with docking scores <–150 and confidence scores >0.7 were considered potential interactors. To further validate interaction potential, complex structures were predicted using AlphaFold3. Each candidate was modeled in complex with ASFV I215L, and model reliability was evaluated using the predicted template modeling score (pTM) and the interface predicted template modeling score (ipTM). A pTM > 0.5 was regarded as supportive of a plausible global fold, while an ipTM > 0.6 indicated a reliable subunit arrangement. A composite ranking score integrating ipTM, pTM, disordered fraction, and steric clashes was also calculated.

### Structural modeling and mutational analysis

The predicted I215L–FAM105A complex was visualized in PyMOL (version 3.1.0; Schrödinger, LLC). Models were color-coded according to pLDDT confidence scores, and interface residues within 4 Å were identified, with polar contacts highlighted. To assess the contribution of individual residues, seven polar contact residues in I215L (E19, N20, M99, S102, A104, D108, and S116) were subjected to mutational scanning using FoldX (version 5.0). Each residue was systematically substituted with the other 19 amino acids, and the resulting changes in binding free energy (ΔΔ*G*) were calculated. Positive ΔΔ*G* values indicated destabilizing mutations, whereas negative values indicated stabilizing effects.

### Plasmid construction

The pHAGE vector with 3×Flag or 3×Myc plasmid backbone was constructed using plasmids previously generated in our laboratory (Yang *et al.* 2024). The plasmid Phage-3×Flag-I215L was constructed by cloning the synthesized ASFV I215L (GENEWIZ, Suzhou, China) into the phage vector phage-BSD-3×Flag, and its sequence information is shown in supplementary Table S2. The plasmid Phage-3×Myc-FAM105A is the one that has cloned the synthesized pig FAM105A sequence (GENEWIZ, Suzhou, China) into the vector phage-bsd-3×Myc, and its sequence information is shown in supplementary Table S2. All plasmids were constructed using the One-Step Cloning Kit (Vazyme, Nanjing, China). DNA sequencing confirmed the desired specific sequences in the constructs (Tsingke, Xi’an, China).

### Cell culture

HEK293T cells were obtained from the American Type Culture Collection (CRL-11268) and were cultured in Dulbecco’s Modified Eagle Medium (DMEM, Gibco, #C11995500BT), containing 10% FBS and penicillin (50 U/mL) & streptomycin (50 μg/mL) at 37°C in a humidified atmosphere of 5% CO_2_.

### Cell transfection and immunoprecipitation

HEK293T cells were seeded on day 0 at 5 × 10^5^ cells in 6-well plates. The cells were transfected with Phage-3×Flag-I215L and Phage-3×Myc-FAM105A (1000 ng/well) using Polyplus (101000046, Polyplus Transfection, France). At 36-h post-transfection, cells were washed with PBS and then lysed in IP lysis buffer.

For immunoprecipitation analysis, the HEK293T cells were homogenized with IP lysis buffer (50 mmol/L Tris-Cl pH = 7.4, 150 mmol/L NaCl, 1% Triton-100, 0.1% SDS, 1% Sodium deoxycholate, and protease inhibitor cocktail), centrifuged to remove cell debris, and then the supernatant was collected for immunoprecipitation with Anti-Myc (HY-K0206, MCE, USA) or Anti-Flag magnetic beads (HY-K0207, MCE, USA) at 4°C for 2 h. After washing five times with IP lysis buffer, the protein-bound beads were finally resuspended in SDS-PAGE loading buffer. The samples were boiled at 95°C for 10 min, and the supernatant was loaded on the gel for immunoblotting.

## RESULTS

### Computational identification of I215L interactors

The overall workflow for identifying host proteins interacting with ASFV I215L is depicted in Fig. 1. First, 456 swine ubiquitin thioesterase sequences were retrieved from UniProt. After removing duplicates and retaining entries with reviewed annotations and available 3D structures, 34 non-redundant candidates were selected for further analysis. These candidates were subjected to a two-step computational pipeline: HDOCK protein−protein docking to predict potential interactions, followed by AlphaFold3 structural modeling to evaluate the plausibility of the predicted complexes. This integrative approach combines docking affinity and structural reliability to systematically identify host proteins most likely to interact with I215L.

**Figure 1:**
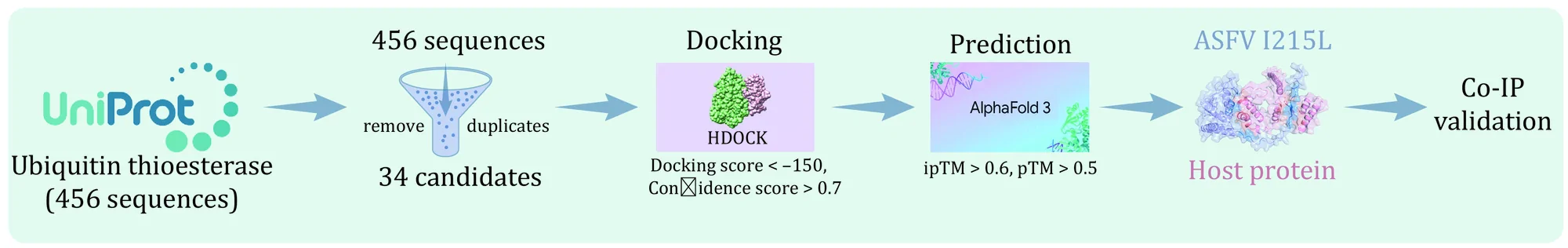
Workflow for identification of ASFV I215L interactors. A total of 456 deubiquitinating enzymes (also known as ubiquitin thioesterases) protein sequences were retrieved from UniProt. After removing duplicates and unreviewed entries, 34 unique candidates were retained for computational screening

In HDOCK, the docking score reflects predicted binding propensity, with more negative values indicating more favorable binding models, while the confidence score, derived from the docking score, indicates the likelihood of interaction. Scores above 0.7 suggest a highly probable interaction (Yan *et al.* 2020). Of the 34 candidates, 20 met the predefined thresholds (docking score < −150 and confidence score > 0.7) (Fig. 2A). Among these, USP21, a well-characterized deubiquitinase involved in the regulation of immune signaling and protein homeostasis (An *et al.* 2022), showed the strongest predicted interaction (docking score = −313.51, confidence score = 0.96). FAM105A, an OTULIN-like thioesterase (Weinelt and van Wijk 2021), also ranked highly (docking score = −271.92, confidence score = 0.92), suggesting a novel link between ASFV I215L and host deubiquitinating machinery.

**Figure 2:**
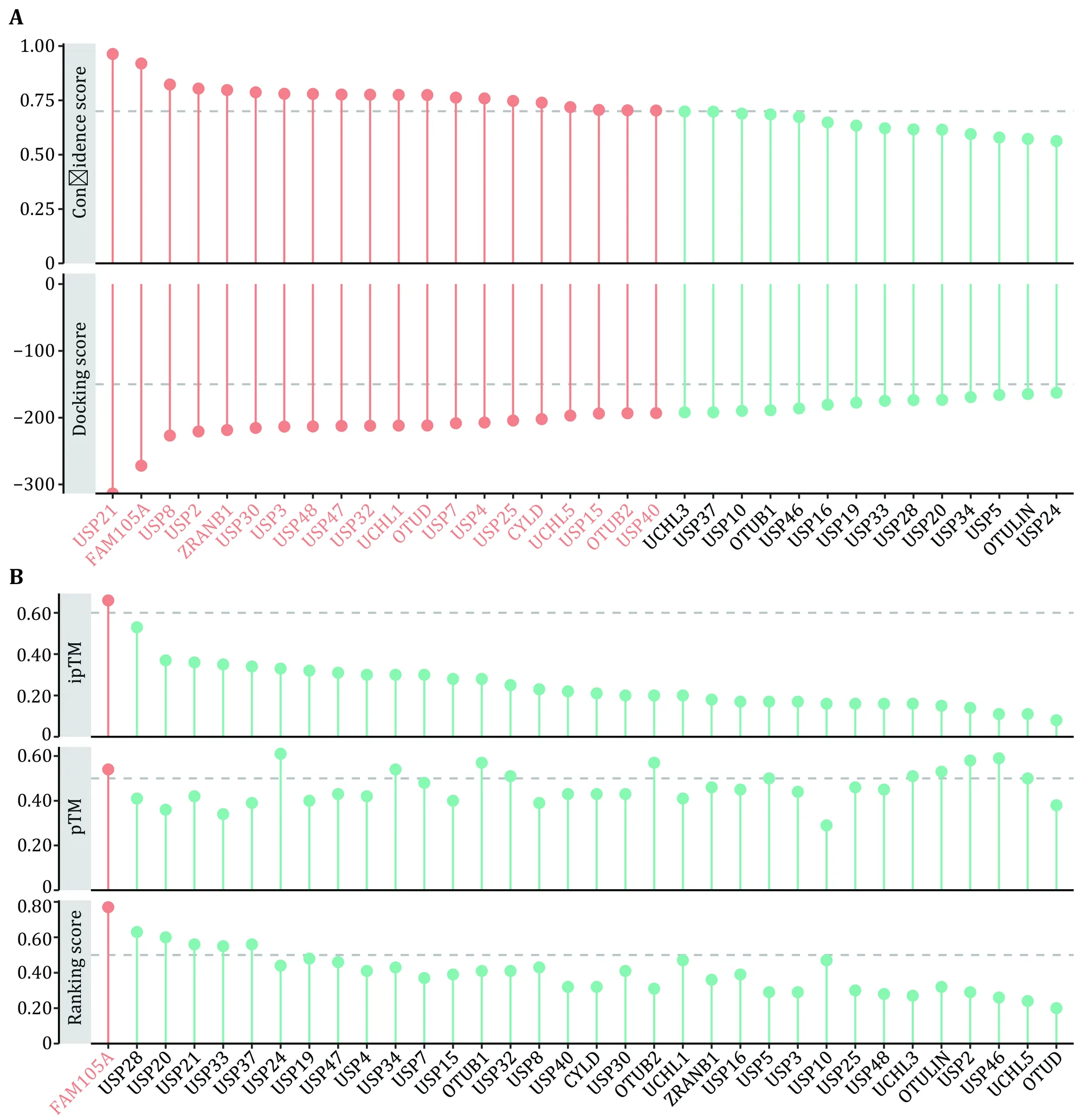
Computational screening of candidate deubiquitinating enzymes for interaction with ASFV I215L. **A** HDOCK docking results. Twenty-one proteins pass the thresholds (docking score < –150, confidence score > 0.7), with USP21 and FAM105A showing the strongest predicted interactions. **B** AlphaFold3 complex prediction. Only FAM105A meets the structural reliability criteria (ipTM = 0.66, pTM = 0.54, ranking score = 0.77), supporting its identification as the most probable I215L interactor

AlphaFold3 was then used to model the 3D structures of candidate complexes. Model quality was assessed using the predicted TM-score (pTM), which reflects overall structural reliability (pTM > 0.5 indicates a plausible structure); the interface predicted TM-score (ipTM), which indicates confidence in the interaction interface (ipTM > 0.6 denotes a reliable interface); and an integrated ranking score combining pTM, ipTM, fraction of disordered residues, and steric clashes, with higher scores representing more reliable models (Abramson *et al*. 2024). Among all candidates, only FAM105A satisfied these structural reliability criteria (pTM = 0.54, ipTM = 0.66, ranking score = 0.77), confirming it as the most probable I215L interactor (Fig. 2B). Collectively, these results establish FAM105A as the top computationally predicted binding partner for ASFV I215L, based on both docking affinity and structural plausibility.

### Experimental validation and interface analysis of the I215L–FAM105A interaction

To experimentally validate the computational predictions, co-immunoprecipitation (Co-IP) assays were performed in HEK293T cells. Reciprocal pulldown using differently tagged constructs confirmed a physical interaction between I215L and FAM105A (Figs. 3A and 3B), providing direct support for the computational predictions.

**Figure 3:**
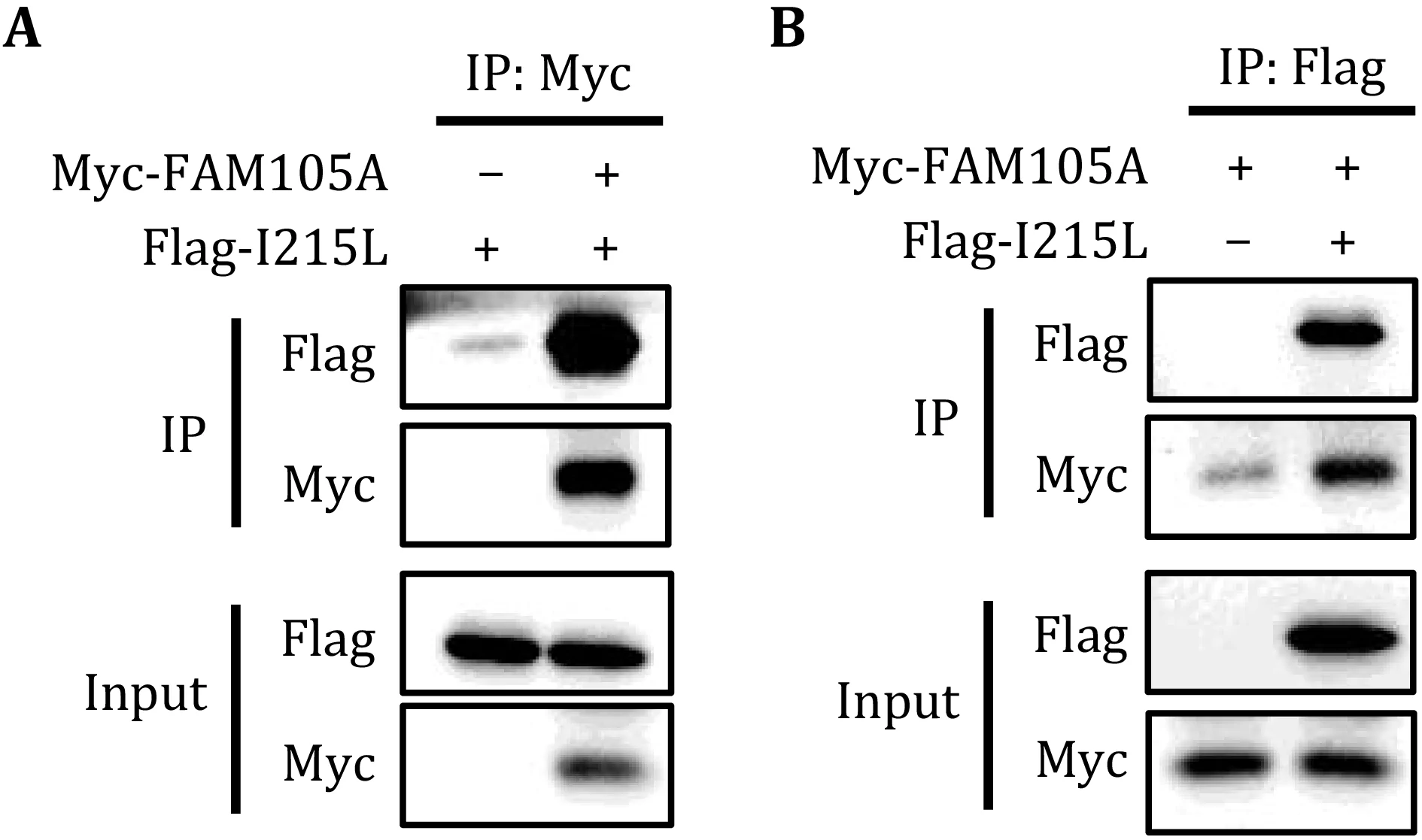
Verification of the interaction between I215L and FAM105A. **A**,**B** Co-immunoprecipitation (Co-IP) assays confirming the interaction between I215L and FAM105A using reciprocal tag pulldown in HEK293T cells

To define the molecular basis of this interaction, the AlphaFold3-predicted I215L–FAM105A complex was analyzed. The predicted complex exhibited well-defined interface regions with high local confidence (pLDDT) (Fig. 4A). Seven I215L residues — E19, N20, M99, S102, A104, D108, and S116 — were positioned within 4 Å of FAM105A, forming specific polar contacts (E19–K166, N20–E169, M99–N178, S102–S193, A104–N194, D108–R200, S116–C177) (Fig. 4B).

**Figure 4:**
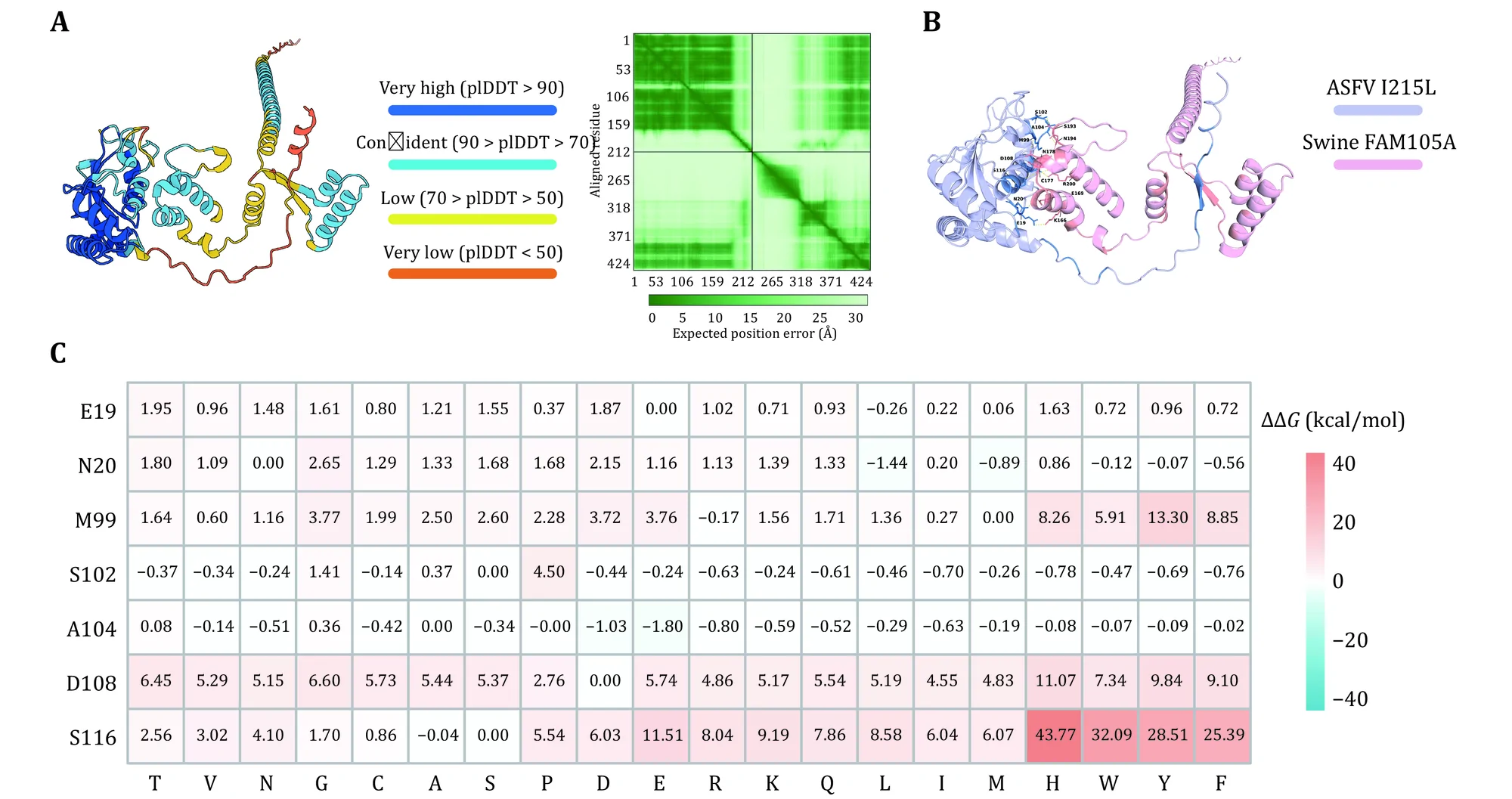
Mutational analysis of the ASFV I215L–FAM105A interface. **A** AlphaFold3-predicted complex structure, colored by pLDDT confidence scores. **B** Key polar residues on I215L (E19, N20, M99, S102, A104, D108, S116) are within 4 Å of FAM105A. **C** FoldX mutational scanning shows that substitutions at these residues significantly alter binding free energy (ΔΔ*G*), highlighting their role in stabilizing the interaction

The contributions of these residues to complex stability were quantitatively assessed using FoldX mutational scanning. FoldX provides rapid and accurate predictions of the effects of point mutations on protein and complex stability based on full-atomic energy calculations derived from empirical protein engineering data (Delgado *et al.* 2019). Substitutions at any of the seven residues markedly altered the binding free energy (ΔΔ*G*) (Fig. 4C), confirming their importance in stabilizing the interaction. Among them, M99, D108, and S116 had the most pronounced effects. For instance, mutations at S116 resulted in ΔΔ*G* values of 25.39 kcal/mol (F), 28.51 kcal/mol (Y), 32.09 kcal/mol (W), and 43.77 kcal/mol (H), highlighting its critical role in maintaining complex stability.

## DISCUSSION

African swine fever virus (ASFV) remains a major threat to global pig production due to its high mortality and the lack of effective vaccines or antiviral therapies (Li *et al.* 2022; Zhang *et al.* 2023). Viral manipulation of host ubiquitin pathways is a critical mechanism by which ASFV evades immune defenses and promotes replication (Freitas *et al.* 2018; Huang *et al.* 2021; Lin *et al.* 2024). Affinity purification-mass spectrometry (AP-MS) is one of the most widely used methods for mapping protein interactions on a large scale (Bludau and Aebersold 2020). However, it is costly and time-consuming, particularly for the large DNA viruses like ASFV, which encodes over 150 proteins (Pérez-Núñez *et al.* 2019). Here, we systematically screened 34 swine DUBs using HDOCK docking and AlphaFold3 modeling, and identified FAM105A as a novel interactor of ASFV I215L. OTULIN (also known as FAM105B), a member of the ovarian tumour (OTU) deubiquitinases (DUBs) family, specifically removes linear polyubiquitin chains from substrates and acts as a regulator of innate immune response (Begalli *et al.* 2017). FAM105A, also known as OTULINL, shares a low amino acid sequence identity with OTULIN and lacks catalytic function against all ubiquitin linkages (Weinelt and van Wijk 2021). Although very little is known about the cellular and biochemical function of FAM105A, its subcellular ER membrane localization and interactome suggest that it may play a role in ER-organelle communication (Ceccarelli *et al.* 2019). ER is essential for ASFV replication and maturation, and ASFV infection activates the UPR response, which in turn facilitates viral replication (Galindo and Alonso 2017). As an early expression gene, I215L plays a key role in ASFV infection (Freitas *et al.* 2018). Although we successfully identified FAM105A as an interactor of I215L by integrative computational predication and experimentally validation, the biological significance of this interaction, particularly in relation to ER stress/UPR and ASFV replication, requires further investigation. Furthermore, as an E2 enzyme, I215L recruits some substrates to undergo ubiquitination and subsequent proteasomal degradation (Huang *et al.* 2021; Riera *et al.* 2023). Future research should also investigate the ubiquitination and stability of FAM105A when interacting with I215L.

In conclusion, our integrative approach, which combines docking, deep-learning-based structural modeling, and mutational scanning, provides a robust framework for identifying virus–host protein interactions and for characterizing residues critical for maintaining binding stability. This strategy can be applied to other viral proteins, facilitating the discovery of novel host targets and guiding rational antiviral development.
